# ScbR- and ScbR2-mediated signal transduction networks coordinate complex physiological responses in *Streptomyces coelicolor*

**DOI:** 10.1038/srep14831

**Published:** 2015-10-07

**Authors:** Xiao Li, Juan Wang, Shanshan Li, Junjie Ji, Weishan Wang, Keqian Yang

**Affiliations:** 1 State Key Laboratory of Microbial Resources, Institute of Microbiology, Chinese Academy of Sciences, Beijing, 100101, People’s Republic of China

## Abstract

In model organism *Streptomyces coelicolor*, γ-butyrolactones (GBLs) and antibiotics were recognized as signalling molecules playing fundamental roles in intra- and interspecies communications. To dissect the GBL and antibiotic signalling networks systematically, the *in vivo* targets of their respective receptors ScbR and ScbR2 were identified on a genome scale by ChIP-seq. These identified targets encompass many that are known to play important roles in diverse cellular processes (e.g. *gap1*, *pyk2*, *afsK*, *nagE2*, *cdaR*, *cprA*, *cprB*, *absA1*, *actII-orf4*, *redZ*, *atrA*, *rpsL* and *sigR*), and they formed regulatory cascades, sub-networks and feedforward loops to elaborately control key metabolite processes, including primary and secondary metabolism, morphological differentiation and stress response. Moreover, interplay among ScbR, ScbR2 and other regulators revealed intricate cross talks between signalling pathways triggered by GBLs, antibiotics, nutrient availability and stress. Our work provides a global view on the specific responses that could be triggered by GBL and antibiotic signals in *S. coelicolor*, among which the main echo was the change of production profile of endogenous antibiotics and antibiotic signals manifested a role to enhance bacterial stress tolerance as well, shedding new light on GBL and antibiotic signalling networks widespread among streptomycetes.

Microorganisms in the nature environment are overwhelmed with diverse chemicals and are engaged in extensive interactions with their community to modulate gene expression^1^. Chemical signalling has been shown to affect phenotypes significantly as diverse as differentiation, antibiotic production, biofilm formation and pathogenicity^2^,^3^,^4^. *Streptomyces* are important bacteria by producing a variety of active secondary metabolites and as one of the model systems for bacterial morphological development. Small molecule signals, such as γ-butyrolactones (GBLs) and antibiotics, have been reported to play vital roles in coordinating secondary metabolism and morphological development in *Streptomyces* species^5^,^6^. To understand these signalling pathways, many studies have been carried out by probing the functions of their signal receptors^3^,^7^.

In the model organism *Streptomyces coelicolor* A3(2), ScbR was characterized as a receptor of GBLs synthesised by the product of the *scbA* gene^5^, while ScbR2 was identified as a receptor of antibiotics^6^. The *scbR*, *scbR2* and *scbA* genes are located in the *cpk* cluster, which determines production of the polyketide coelimycin P1^8^. ScbR binds the *scbR*-*scbA* intergenic region to repress *scbR* while activating *scbA* in response to GBLs^5^. Another known target of ScbR is *kasO*, the pathway-specific activator gene of the *cpk* cluster^9^. Despite its high degree of homology (50%) with ScbR, ScbR2 does not bind GBL molecules, and was previously described as a “pseudo-GBL receptor”^10^. In fact, it binds and responds to endogenous antibiotics, actinorhodin (Act) and undecylprodigiosin (Red), as well as exogenous antibiotics, such as angucyclines^6^. Our previous work demonstrated that angucyclines affect the behavior of *S. coelicolor* by modulating the interaction of ScbR2 with *adpA* (encoding the master regulator of morphogenesis) and *redD* (the direct activator gene of the *red* cluster for Red production)^6^. Neither *adpA* nor *redD* is a target of ScbR. The *scbR2* mutant displayed a complete loss of production of Act, Red and the calcium-dependent antibiotics (CDA), and showed precocious development of aerial hyphae^6^,^10^. These phenotypic effects are much more pronounced than those of the *scbR* mutant, which mainly showed delayed Red production^5^. Interestingly, ScbR2 also binds the same sites as ScbR in the *scbR*-*scbA* intergenic region to shut down GBL synthesis and in the promoter region of *kasO* to control the production of coelimycin P1^10^,^11^. How do the signalling pathways mediated by ScbR and ScbR2 interplay and cooperate in eliciting specific physiological responses of *S. coelicolor* is largely unknown. Moreover, homologs of ScbR and ScbR2 are widespread among streptomycetes, but studies regarding to them were all focussed on the regulation of genes for antibiotic biosynthesis^12^,^13^. In order to gain a more comprehensive overview of the roles of such receptors, we have undertaken a genome-wide analysis of the regulatory targets of ScbR and ScbR2 in *S. coelicolor*.

In this work, targets of ScbR and ScbR2 were deciphered using chromatin immunoprecipitation followed by sequencing (ChIP-seq) and combined with transcriptomic expression analysis. These targets were engaged in diverse physiological processes, but a major role of them was to control secondary metabolism and elicit stress responses. Furthermore, ScbR and ScbR2 mediated regulatory cascades, feedforward loops (FFLs) and sub-networks were extracted to control *Streptomyces* phenotypes. The interplay among ScbR, ScbR2 and other regulators revealed intricate cross talks between signalling pathways triggered by GBLs, antibiotics, nutrient availability and stress.

## Results

### Mutational analysis and expression profiles of *scbR* and *scbR2* in *S. coelicolor*

It was previously reported that an *scbR* mutant showed delayed Red and reduced Act production^5^. For further analysis, we constructed an in frame mutant of *scbR* in *S. coelicolor* M145. In ΔscbR, a delay of aerial development was noted after growing for 36 h on SMMS plates (Supplementary Fig. S1a). Red and Act in ΔscbR were produced almost synchronously with M145, but their production levels were greatly reduced (Supplementary Fig. S1bc). A similar pattern of production was observed for CDA: a plate-based bioassay of CDA production revealed a dramatic decrease of CDA level in ΔscbR (Supplementary Fig. S1d). Moreover, a yellow-pigmented secondary metabolite was observed in ΔscbR on SMMS plate, which was probably due to the production of coelimycin P1, as also reported for ΔscbR2 (also known as scbR2DM), in which coelimycin P1 was abundantly synthesised^8^,^14^ (Supplementary Fig. S1a). In comparison, ΔscbR2 showed a much more striking phenotype of complete loss of Act, Red and CDA production^10^ and precocious formation of aerial hyphae^6^. These observations demonstrated the pleiotropic effects of ScbR and ScbR2 on the physiology of *S. coelicolor*.

**Figure 1:**
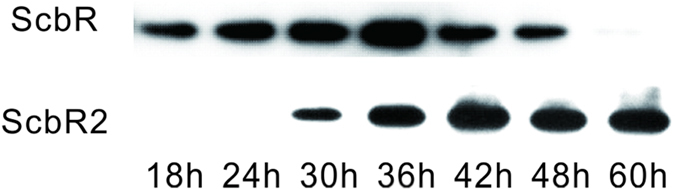
Protein levels of ScbR and ScbR2 during growth period. Western blotting was carried out to analyse the temporal expression of protein ScbR and ScbR2 during growth in liquid medium.

Before deciphering regulons of ScbR and ScbR2, their expression profiles at transcript and protein levels were monitored in SMM liquid culture. Our previous work established that transcription of *scbR* began at 24 h and peaked at 36 h^11^. In concert, ScbR protein was detected at 24 h by western blotting, and accumulated to the highest level at 36 h (Fig. 1). ScbR can be dissociated from its targets by SCB1, which is synthesized by ScbA, the mRNA for which also reached the highest level around 36 h^11^. Therefore, 30 h was chosen as the sampling time point for the ScbR ChIP experiment, when the level of ScbR protein was considerable but the transcript of *scbA* had not reached the highest level. In contrast, *scbR2* transcript was not apparent until 36 h, peaked at 48 h and maintained a relatively high level until 72 h^11^. Also, ScbR2 protein could barely be detected at the beginning of growth (18 or 24 h), rose to the highest level at 42 h, and remained stable to 60 h (Fig. 1). ScbR2 can be dissociated from its targets by the endogenous antibiotics, Red and Act^10^. Act appeared in M145 after 48 h, while Red accumulated after 36 h (Supplementary Fig. S1bc). Therefore, 42 h was chosen as the sampling time point for the ScbR2 ChIP experiment, when ScbR2 protein was at the highest level and the production of Act and Red was still relatively low.

### Overview of ChIP-seq and transcriptomic profiling results

**Figure 2:**
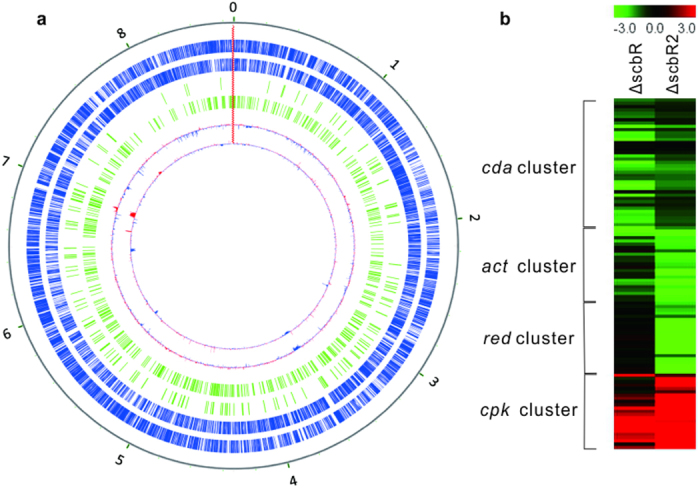
Distribution and attributes of global targets of ScbR and ScbR2. (**a**) A map of the *S. coelicolor* genome with ChIP-seq of ScbR and ScbR2 and transcriptome profiling data of their mutants. From the outmost to the innermost, coding sequences in the genome are shaded in blue as the two outer circles, the genomic distribution of ScbR and ScbR2 targets are shown in green. The transcriptome profiling of scbR and scbR2 mutants are indicated in the innermost two circles: the red lines represented genes up-regulated by mutation, while the blue indicated down-regulated genes. (**b**) Transcriptional effects of ScbR and ScbR2 on four antibiotic cluster genes. Red indicates activation; green indicates repression, while dark means no change.

ChIP-seq experiments of ScbR and ScbR2 were performed with purified antibodies in *S. coelicolor* M145 in liquid SMM culture as previously described^6^. As a control, sheared chromosome DNA (input DNA) was utilised to subtract background noise. On ChIP-seq maps, the fold change of peaks above 1.5 was fixed as the minimum cut-off value for ScbR and ScbR2 peak calling. On this basis, 144 ScbR peaks and 491 ScbR2 peaks were detected, distributed relatively even along the chromosome (Fig. 2a). Grouped together by the clustering algorithm MACS^15^, many of these significant peaks encompassed multiple genes and almost equally occurred in protein-coding and non-coding regions. The peaks generated included the majority of promoter regions known to be targeted by ScbR and ScbR2, i.e. *scbR*-*scbA* and *kasO* promoters were detected among the peaks of ScbR; *scbR*-*scbA*, *kasO* and *adpA* promoters were noted among peaks of ScbR2, suggesting the reliability of ChIP-seq.

To assess the transcriptional effects of ScbR and ScbR2 binding on targets genes, a genome-wide transcriptomic analysis of the parent strain M145 and the *scbR* or *scbR2* mutants was performed. RNA samples were harvested at 30 h from ΔscbR and at 42 h from ΔscbR2, and they were analysed on an Agilent based microarray platform. 42.3% of genes demonstrated at least 20% difference in expression levels in ΔscbR, while 30.1% of genes showed greater than 20% change in ΔscbR2, underscoring the pleiotropic influence of the two regulators. Transcriptional changes of antibiotic synthesis gene clusters were consistent with phenotypes we observed (Supplementary Fig. S1). In ΔscbR, transcription of *act* and *cda* cluster genes was dramatically reduced, while *cpk* cluster genes were activated, but the mRNA level of *red* cluster genes decreased only slightly (Fig. 2b). Similarly, *act*, *red* and *cda* gene cluster mRNA was much less abundant in ΔscbR2, but *cpk* cluster mRNA was greatly enhanced.

### *In vitro* confirmation of ScbR or ScbR2 binding events on selected promoter regions

To validate the results from ChIP-seq, the binding of ScbR and ScbR2 was analysed by electrophoretic mobility shift assays (EMSA). Peaks locating in the coding regions was first excluded, and attention was focused on regulation occurred in promoter regions (−600 ~ + 100 relative to the putative translational start point), especially those associated with genes that were well studied or vitally annotated were chosen for further validation (Supplementary Fig. S2). Thus, 23 targets of ScbR and 76 targets of ScbR2 (Supplementary Table S1 and S2) were chosen for evaluation. Recombinant ScbR or ScbR2 proteins were purified from *E. coli* and EMSA assays were performed with their selected promoter probes as described before^11^. In total, 7 out of 23 targets of ScbR, and 40 out of 76 targets of ScbR2 were newly confirmed to show direct binding (Table 1 and 2), among which five common targets could interact with both ScbR and ScbR2. According to our previous work^11^, ScbR and ScbR2 have identical binding sites; therefore we expected that ScbR and ScbR2 could also each bind newly identified targets of the other. EMSA experiments were then performed with ScbR protein and targets of ScbR2, and vice versa. It turned out 6 targets from ScbR2 were able to bind with ScbR, and the rest two targets of ScbR could also interact with ScbR2. In summary, 13 and 42 promoters were confirmed by EMSA to be bound by ScbR (Table 1) and ScbR2 (Table 2), respectively. Failure to detect those targets by ChIP was probably due to the competitive binding between ScbR and ScbR2, or to the use of different time points in the two ChIP experiments. Surprisingly, ScbR only bound a fraction of ScbR2 targets whereas ScbR2 bound to all ScbR targets. 69.2% of ScbR target genes and 58.1% of ScbR2 being transcriptionally activated or repressed (fold change > 1.2 or <0.85) (Table 1 and 2). Among those EMSA confirmed targets, we found the binding events did not necessarily lead to changes in expression of adjacent genes. This may be due to the need of assistant proteins to fulfil their roles, or to particular time points and culture conditions selected.

**Figure 3:**
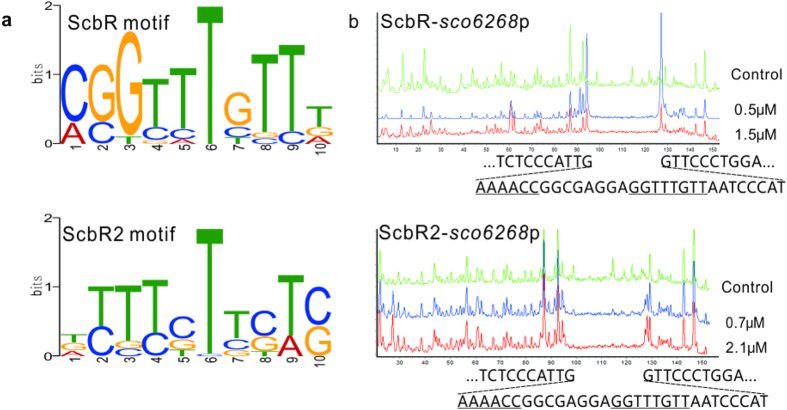
Conserved motifs and DNase I footprinting of ScbR and ScbR2 on *sco6268* promoter. (**a**) Conserved motifs of ScbR and ScbR2. All binding sequences of ScbR or ScbR2 were submitted to MEME algorithm for motif derivation. (**b**) Binding sites of ScbR and ScbR2 on *sco6268* promoter. Footprints of ScbR and ScbR2 are shown between dashed lines, and the MEME predicted motifs were underlined.

To gain further insights into the binding sites of ScbR or ScbR2, all binding sequences of ScbR or ScbR2 were submitted to the MEME algorithm^16^. A10-nt conserved motif 5′-MSGYTTSTTD-3′was derived for ScbR and a 5′-DYTYSTYSWS-3′ for ScbR2, respectively, as shown in Fig. 3a, resembling the consensus previously extracted from limited sequences^9^,^11^. The two motifs overlapped at a relatively conserved 5′-TTTSTT-3′ element, and overall, ScbR showed a higher specificity than ScbR2. The motif derived from exclusive targets of ScbR2 was almost the same as the motif derived from all target sequences of ScbR2 (data not shown), implying those ScbR2-specific binding sequences are more degenerate, and this degeneracy prevented recognition by ScbR that preferred more conserved binding sequences. Comparison of these two motifs could explain why ScbR2 is capable of binding to all ScbR targets while ScbR only binds a fraction of ScbR2 targets.

To validate the MEME-predicted binding motifs, recombinant ScbR and ScbR2 were further used to conduct DNase I footprinting analysis as described before^11^, on the HEX dye labelled promoter region of *sco6268*, which was shown to bind with both ScbR and ScbR2 (Supplementary Fig. S3). Footprints of ScbR and ScbR2 on *sco6268*p covered the same 30 bp sequences, encompassing two mutually inverted 5′-TTTGG-3′ copies (Fig. 3b), resembling the conserved common element of ScbR and ScbR2 motifs (Fig. 3a). To predict the binding sites of ScbR and ScbR2 on those promoters more precisely, promoters with highly conserved palindromic motifs (*scbA*, *kasO* and *sco6268* promoters), were then used as references for binding site extraction using the MEME algorithm, and the predicted binding sites were shown in Table 1 and 2. Most predicted binding sequences contained two copies of sequences similar to the 5′-TTTGG-3′ motif, but only one of the copies was highly conserved in some sequences. In some promoters, mainly those targeted by ScbR2, only half of the palindrome could be detected. This suggests a reliance on assistant proteins^17^ or a need for DNA configuration change to bring the halves closer.

### Targets of ScbR and ScbR2 in secondary metabolism and development

When analysing the function of their targets, a predominant role of ScbR and ScbR2 was to regulate secondary metabolism both directly and via regulatory cascades and loops. By direct targeting, they both interacted with the promoter of *sco6268* (which encodes a histidine kinase) in the *cpk* cluster, in addition to the promoter of *kasO*^9^,^10^, to repress coelimycin P1synthesis. As a result, most genes from the *cpk* cluster were transcriptionally activated in the *scbR* and *scbR2* mutants (Fig. 2b). Since ScbR and ScbR2 were observed to be expressed during different time periods, single mutant of each regulator could exert a regulatory effect on *cpk* synthesis. Also, ScbR and ScbR2 influenced CDA synthesis by binding to the promoter of the activator gene, *cdaR*; as expected, most genes of the *cda* cluster were down-regulated in both ΔscbR and ΔscbR2 mutants (Fig. 2b). In this case, binding appears to activate, rather than repress, expression of the pathway activator gene. In addition, ScbR and ScbR2 may also exert an effect on reducing power supply for antibiotic production by targeting the expression of *soyB1*, which encodes a ferredoxin pivotal in transferring electrons to cytochrome P450 genes for secondary metabolism^18^; *soyB1* was down-regulated in both ΔscbR and ΔscbR2 (0.611 and 0.613 fold, respectively).

**Figure 4:**
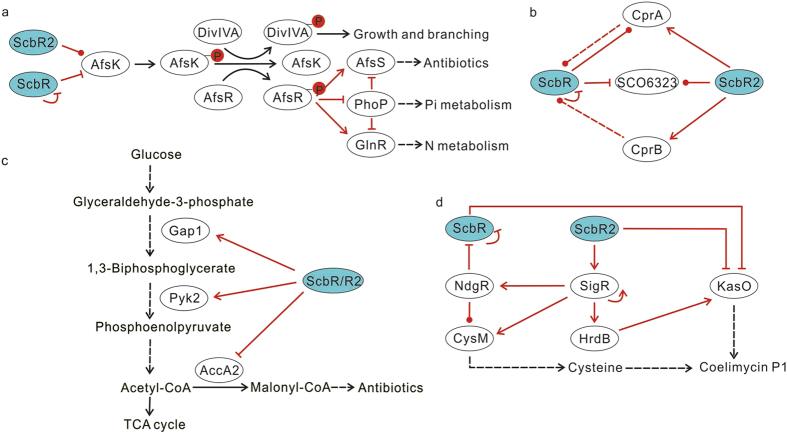
ScbR and ScbR2 mediated regulatory cascades and sub-networks. (**a**) The involvement of AfsK in the regulatory cascades from ScbR or ScbR2 to growth or branching, antibiotic production and nutrition metabolism. (**b**) A complex regulatory network among GBL receptor homologues in *S. coelicolor.* (**c**) Control on the glycolysis and carbon flow by ScbR and ScbR2. (**d**) A sub-network stemming from the regulation of ScbR2 on sigma factor SigR. Regulatory interaction is indicated in red, while metabolite flow is indicated in black. Line with arrow represents activation, with bar represents repression, while with dot means the regulatory effect is unclear or dual function.

On the other hand, ScbR and ScbR2 made up the first step in some regulatory cascades (Fig. 4a). One case involved AfsS, target of the AfsKR two component system^19^ and was proposed to relate GBL signalling to the Act and Red production phenotypes^20^. Here, we further support this idea by identifying *afsK* as a target of ScbR and ScbR2. In ΔscbR, consistent with the diminished production of Act and Red, expression of *afsS* was greatly reduced (by 0.317 fold). Remarkably, AfsK is also implicated in polar growth and hyphal branching by phosphorylating DivIVA, and high AfsK activity could cause growth impediment^21^. This suggested a correlation between the phenotype of growth arrest (Supplementary Fig. S1) and the enhanced *afsK* expression (1.405 fold) in ΔscbR.

Thirdly, a sub-network involving ScbR and ScbR2 regulation was revealed. The promoter of *cprA*, which encodes another close homologue of ScbR, was bound by ScbR and ScbR2, and *cprB*, also encoding a close homologue of ScbR, was a ScbR2 target. Both CprA and CprB were reported to be involved in the regulation of antibiotic production and sporulation^22^, and recently we found they repress GBL synthesis by binding to the promoter of *scbA* (unpublished results). SCO6323, encoding another ScbR homologue in *S. coelicolor*, was also demonstrated here to be under the control of ScbR and ScbR2. Therefore, a complex regulatory network among GBL receptor homologues in *S. coelicolor* intervenes to control complex phenotypes (Fig. 4b).

Besides the common targets with ScbR, the antibiotic receptor ScbR2 appears to exert much more profound control on secondary metabolism. As a cluster-situated-regulator (CSR), ScbR2 bound more promoter regions within the *cpk* cluster. In addition to *kasO* and *sco6268* promoters, it also bound at the intergenic regions of *sco6271*-*sco6272*, *sco6275*-*6276*, and *sco6282*-*sco6283*, and the promoter of another regulatory gene *sco6288*. Similarly, in the *cda* cluster, beside the CSR gene *cdaR*, it also targeted the promoters of structural genes *sco3229-sco3230* and *sco3249*. Most strikingly, Act and Red production were completely abolished in ΔscbR2, a phenotype partly explained by the direct regulation by ScbR2 of the corresponding CSR genes *actII-orf4*, *redD* and *redZ*^23^,^24^. Transcription of them was greatly reduced in ΔscbR2 (Table 2). Furthermore, ScbR2 directly regulates the pleiotropic regulatory genes *atrA*^25^ and *absA1/A2*^26^ to control antibiotic production. *atrA* was repressed (0.563 fold) and *absA1/A2* was induced (Supplementary Fig. S4) in ΔscbR2. Overall, about one third of targets of ScbR2 were found to affect antibiotic biosynthesis, revealing a key role of ScbR2 in the control of antibiotic production phenotypes.

### Targets of ScbR and ScbR2 involved in primary metabolism and other processes

The involvement of ScbR and ScbR2 in primary metabolism was mainly observed at three critical nodes in carbon flow, nitrate respiration and acetylglucosamine (GlcNAc) transport. Two genes encoding enzymes in glycolysis, *gap1*and *pyk2* (the former encodes a glyceraldehyde-3-phosphate dehydrogenase responsible for the synthesis of 1,3-biphosphoglycerate from glyceraldehyde -3 phosphate; the latter encodes a pyruvate kinase controlling an irreversible reaction from phosphoenolpyruvate to pyruvate), were found as targets of ScbR and ScbR2 (Fig. 4c). Reduced transcription of *gap1* in ΔscbR and ΔscbR2 (0.650 and 0.768 fold) suggests a slowdown of primary metabolism. Although, transcription of *pyk2* was marginally changed in ΔscbR, it was reduced in ΔscbR2 (0.783 fold). Another important target gene of ScbR and ScbR2, *sco4921* (*accA2*), encodes the A subunit of acetyl-CoA carboxylase, which is the key enzyme involved in converting acetyl-CoA to malonyl-CoA, thus providing precursors for antibiotic synthesis (notably polyketides such as Act and coelimycin P1) (Fig. 4c). Overexpression of *accA2* in both ΔscbR and ΔscbR2 (1.650 and 2.464 fold) would direct more acyl-CoA flux toward malonyl-CoA for antibiotic production. NarG3, a component of respiratory nitrate reductase^27^, was also found as a target of ScbR and ScbR2, and its expression was induced in both ΔscbR and ΔscbR2 (1.487 and 2.347 fold). ScbR and ScbR2 were also involved in the regulation of nutrition utilization by targeting *nagE2*, which encodes the only permease for GlcNAc, a primary source of carbon and nitrogen for streptomycetes^28^,^29^. As mention above, *afsK* was under direct control from ScbR and ScbR2, downstream its response regulator AfsR was also closely involved with nitrogen and phosphate metabolism by binding with the promoters of the corresponding regulatory genes *glnR* and *phoR*/phoP^30^,^31^, and regulator PhoP could control GlnR and AfsS to correlate nutrition metabolism with antibiotic production^31^,^32^ (Fig. 4a). GBL and antibiotics are therefore involved in nutrition utilization as well.

Also ScbR2 controlled specific targets other than that shared with ScbR. The ribosome is a key node in cell associating with antibiotic-inducing responses and bacterial drug resistance^33^. Surprisingly, ScbR2 ChIP-seq binding peaks were associated with genes encoding ribosomal proteins, *rpsD* and *rpsL*, and were also present inside and upstream of ribosomal RNA genes (Supplementary Fig. S5), showing a role of ScbR2 in controlling ribosome assembly. The ability of a cell to synthesize proteins during stationary phase was thought the indication of its ability to produce secondary metabolite^34^. Accelerated protein synthesis displayed by overexpressed *rpsL* and *rpsD* in ΔscbR2 (1.418 and1.871 fold) could contribute to increased antibiotic production. In streptomycetes, mutants of σ factors implicated in stress-response are also perturbed in antibiotic production^35^. Among ScbR2 targets, we also identified genes for sigma factors. For example, SigR controls the response to thiol-oxidative stress^36^, and maintains the level and activity of the housekeeping sigma factor HrdB^37^. Interestingly, one of the targets of SigR, NdgR, can bind to the *scbR-scbA* intergenic region^38^, so the regulation on SigR resulted in two cascades from ScbR2 to KasO: ScbR2-SigR-NdgR-ScbR-KasO, and ScbR2-SigR-HrdB-KasO (the *kasO* promoter is recognised by the HrdB sigma factor^39^), as shown in Fig. 4d. Also, overexpressed *sigR* in ΔscbR2 (1.620 fold) was speculated to increase cysteine synthesis to meet the high demand of *N*-acetylcysteine in coelimycin P1 biosynthesis^8^ due to induction on cysteine synthesis gene *cysM* by SigR^40^. Three other sigma factors were found as targets of ScbR2: Sig15 was reported to play a role in osmotic stress response^41^; SigF controls late stages of spore development in *Streptomyces*^42^; and SCO4677, an anti-sigma factor (F), was found to repress antibiotic production and morphological differentiation^43^. Through regulation of sigma factors, ScbR2 also intensely relate with stress responses, further supporting a role of ScbR2 in eliciting survival responses in perception of antibiotic signals.

### Refinement of a local regulatory network and FFL motifs

**Figure 5:**
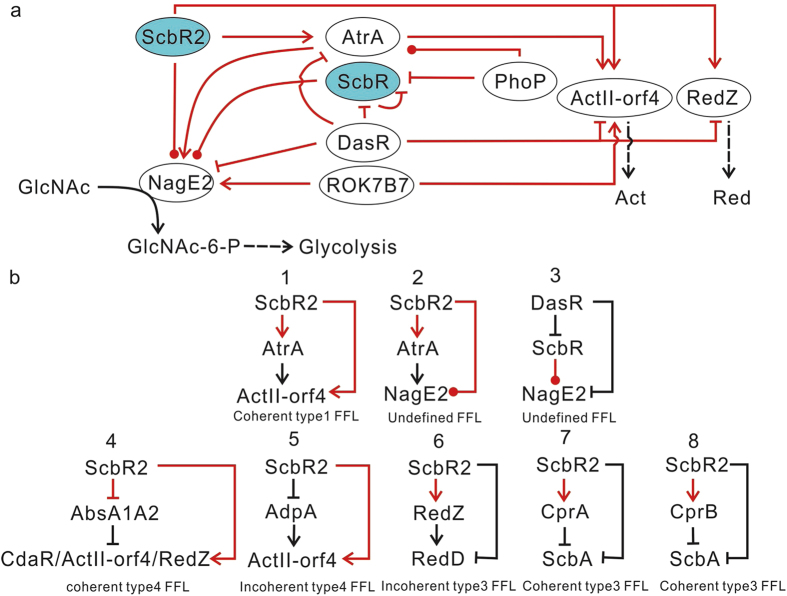
The refinement of a comprehensive sub-network and 10 FFLs. (**a**) A comprehensive sub-network involving control of GlcNAc transport and antibiotics production. Regulatory interaction is indicated in red, while metabolite flow is indicated in black. Lines with arrows represent activation; lines with bars represent repression, while lines with dots mean the regulatory effect is unclear. (**b**) 10 refined FFLs. Red lines represent newly found regulatory interaction.

Besides ScbR and ScbR2 (Supplementary Fig. S3), *nagE2* is also under multi-level regulation including repression by DasR (a master GlcNAc-responsive regulator)^23^,^28^, activation by AtrA (an activator of Act production)^25^, and activation from ROK7B7, which affects xylose utilization and carbon catabolite repression^44^,^45^. Recently, AtrA was reported to be regulated by DasR^23^, thus our work brought in more interplays among these regulators on the control of GlcNAc transport (Fig. 5a). Such interplay also occurred upstream of *actII-orf4*, a known target of DasR, AtrA and ROK7B7^25^,^28^,^46^. Another antagonism happened between ScbR2 and DasR on the *redZ* promoter^28^, further indicating a close relation between GlcNAc transport and antibiotic production (Fig. 5a). Both *actII-orf4* and *atrA* are shown here to be under the direct control of ScbR2 (Supplementary Fig. S3). Thus two FFLs are formed: ScbR2-AtrA-ActII-orf4 and ScbR2-AtrA-NagE2 (Fig. 5b, 1&2). The former is a type I coherent FFL^47^, in which both regulators X and Y activate the expression of gene *Z* while regulator X could also activate expression of regulator Y, but the latter FFL controlling NagE2 is an undefined type, since the regulatory effects of the binding of ScbR2 to NagE2 on the gene expression is statistically insignificant from microarray analysis. ScbR was reported to be down-regulated by DasR^46^, hence another FFL was formed by DasR-ScbR-NagE2 to control the expression of *nagE2* (Fig. 5b 3). ScbR is also regulated by PhoP^17^, which could control the *atrA* expression^17^, indicating a complex interaction among GBL and antibiotic signalling with phosphate nutrition. Based on these interactions, comprehensive linkages were built between primary metabolism and secondary metabolism, nutritional signals (C, N and Pi sources) and signalling molecules (GBL and antibiotics) (Fig. 5a). This local regulatory network also provides a basic picture of regulatory networks and the underlying regulatory mechanisms.

Seven more FFL loops were extracted from the newly identified regulatory interactions (Fig. 5b 4–7). For example, *actII-orf4* was found to be under control of two more FFL motifs via AbsA1A2 or AdpA as mediators^26^,^48^. When *scbR2* is disrupted, AtrA is expected to be down-regulated, but AdpA and AbsA1A2 should be up-regulated (based on the Gus test shown in Supplementary Fig. S4), and the effects of reduced activation by AtrA and ScbR2, increased activation by AdpA, and increased repression from AbsA1A2 would then be integrated at the *actII-orf4* promoter. So the abolishment of Act production in ΔscbR2 may mainly result from a more active AbsA1A2 repression. Likewise, multiple FFLs integration was also found to control expression of *nagE2*, *cdaR*, *redD* and *scbA* and their corresponding phenotypes. Therefore multi-FFLs were employed by *S. coelicolor* to control key cellular events.

## Discussion

In this work, by ChIP-seq and transcriptome analysis, a global view of the specific responses triggered by GBL and antibiotic signalling and the regulatory networks downstream ScbR and ScbR2 were obtained. Unlike the well-studied GBL system of *S. griseus*, in which the GBL receptor ArpA mainly exerts its control by regulating the expression of AdpA that in turn binds and regulates multiple targets^7^, ScbR exerts its effects by directly binding to multiple targets and also binds to targets in primary metabolism that are not found in the GBL regulatory cascades in *S. griseus*. Therefore, the GBL signalling system of *S. coelicolor* is fundamentally different from that of *S. griseus*. ScbR2-mediated antibiotics signalling could provoke large scale physiological responses, including secondary metabolism change, ribosome assembly and induction of stress-related sigma factors. Such responses are beneficial for adaptation and could be vital to the survival of bacteria in their natural habitats. A major response mediated by ScbR and ScbR2 was the shift of endogenous antibiotics production, which could also serve as signals in intra- and interspecies communication or weapons in interspecies competition, implying a role of GBL and antibiotic signalling in streptomycetes ecology.

Interplays between ScbR, ScbR2 and other regulators allow the refinement of complex networks, among which several patterns of regulatory interconnection were extracted. By direct interaction, ScbR2 controls the sequent expression of multiple genes for coelimycin P1 synthesis according to the affinity of ScbR2 with their promoters, a common regulatory node in the metabolic pathway to perform a temporal regulation^47^. Also, ten FFLs involving ScbR and/or ScbR2 were defined in this work. FFLs are important building blocks of regulatory networks^47^. They can generate different phenotypes under different signal strength, as we observed previously with the incoherent FFL consisting of ScbR2-AdpA-RedD^6^, and could also serve other purposes, such as acceleration of signal response and noise filtration^49^,^50^. In the coherent type 1FFL ScbR2-AtrA-ActII-orf4 (Fig. 5b 1), both ScbR2 and AtrA can be deactivated from their targets by Act^10^,^51^. Therefore, when the concentration of Act is low, it would first disassociate the low affinity regulator, but the activation of *actII-orf4* will be maintained by the other activator. Thus the FFL could function to delay and filter the turbulence in Act concentration on *actII-orf4* expression. But when the Act concentration is high enough to disassociate both ScbR2 and AtrA from the *actII-orf4* promoter, the FFL will accelerate the response to shut down the expression of *actII-orf4* and Act production, forming a quick-responsive feedback inhibition mechanism (Fig. 5a). Interestingly, integration of multiple FFLs and utilization of feedback loop were discovered to control GlcNAc transport, antibiotics production, and SCB1 synthesis, which would benefit a stable gene expression^52^,^53^ and permit *S. coelicolor* to show robust adaptation to stimulus. Hence various strategies are used by *S. coelicolor* to adapt to chemical signals and to deal with fluctuating conditions in different environments.

Complex cross-talks between nutrient, stress, GBL and antibiotic signalling pathways were discovered in this work, involving interplays among ScbR, ScbR2 and many key regulators. Some same regulators were found to control both GlcNAc transport and antibiotic production, suggesting a close relation between GlcNAc transport and antibiotic production and the importance of this correlation in the physiology of *S. coelicolor*. GlcNAc is a major nutritional signal for streptomycetes to decide between growth and irreversible sporulation^28^. Therefore to guarantee the accuracy of the decision, multiple regulators, cross-talk between signalling pathways and FFLs formed by these regulators were employed to perform a delicate control on GlcNAc transport. Moreover, cross-talks with other regulators by ScbR or ScbR2, for example, with AdpA, AbsA2, AfsQ1,GlnR, DraR etc at the *actII-orf4* promoter; AbsA2 and AfsQ1at the *cdaR* and *redZ* promoter; and DraR, AfsQ1 and PhoP at the *kasO* promoter^20^, allow the cells to integrate nutritional signals and signals from population growth and environment (manifested by the GBL and Act or Red signals) to adjust the activities of diverse processes, in order to maintain a nutrient homeostasis in natural condition^54^ and to make and support the important decision to sporulate and/or make antibiotics.

## Methods

### Bacterial strains, plasmids, oligonucleotides and growth conditions

Bacterial strains used in this study are listed in the Supplementary Table S3 and the oligonucleotide primers used are listed in Supplementary Dataset S1. *S. coelicolor* strains were incubated on MS solid medium for sporulation and Gus reporter assay. They were grown in liquid SMM medium at 30 °C for western blotting, antibiotic production, ChIP, and microarray experiments. SMMS plates were used for the observation of strain phenotypes. Antibiotic production was detected as described previously^10^. *E. coli* strains were grown in Luria–Bertani medium containing ampicillin (100 μg/mL), kanamycin (50 μg/mL), apramycin (50 μg/mL), hygromycin (50 μg/mL) or chloramphenicol (50 μg/mL) when necessary.

### Western blotting

ScbR and ScbR2 monoclonal antibodies were prepared with recombinant proteins as antigens by CoWin Biotech Co. Ltd as described before^6^. Protein concentration was measured with Bradfold method. Total protein was extracted at 18, 24, 30, 36, 42, 48, 60 hour and 20 μg of each sample was subjected to sodium dodecyl sulfate-polyacrylamide gel electrophoresis (12%). The primary antibodies were diluted at a ratio of 1:3000 from the concentration of 1 mg/ml, while goat anti-rabbit immunoglobulin G- horseradish peroxidase conjugate was used as a secondary antibody at a ratio of 1: 2000.

### Construction of ΔscbR

To construct ΔscbR, a 1903 bp an a 1548 bp homologous arm were amplified from M145 genome with primers scbRLarmF/scbRLarmR and scbRRarmF/scbRRarmR, and digested with HindIII and BamHI, and BamHI and EcoRI, respectively. Digested fragments were then ligated with pKC1139 digested with HindIII and EcoRI to obtain the plasmid pKC1139-∆scbR, which was then conjugated into M145 to obtain the ΔscbR strain. Disruption of *scbR* was verified by PCR, showing that a 502 bp fragment internal to *scbR* gene was deleted.

### ChIP-seq

ChIP experiments were carried out as described previously^6^. Samples of *S. coelicolor* M145 were grown in liquid SMM and harvested at 30 h and 42 h for ScbR and ScbR2 ChIP experiments, respectively. DNA obtained from ChIP experiments was then sonicated into shorter fragments and TruSeqTM DNA Sample Prep Kit-Set A was used to create a pair-end DNA library, which was subsequently amplified with TruSeq PE Cluster Kit and sequenced using Illumina Hiseq2500. MACS^15^ was used to identify peaks of ScbR and ScbR2 binding, and software CGview was used to create and view ChIP-seq maps^55^. Sequencing data were deposited in NCBI Gene Expression Omnibus (GEO, http://www.ncbi.nlm.nih.gov/geo/, number GSE64903).

### Microarray transcriptional profiling

Spores of M145, ΔscbR and ΔscbR2 were inoculated in liquid SMM medium and grown at 30 °C. M145 and ΔscbR were harvested for RNA extraction at 30 h, M145 and ΔscbR2 were harvested at 42 h for RNA extraction. RNAs were then subjected to customized 8*60k Agilent microarray of *S. coelicolor* for hybridization according to the manufacturer’s published protocol (Agilent). Hybridization signals were extracted with Feature Extraction software to obtain raw data, which was introduced into GeneSpring GX software to set parameters and data obtained were then normalised. Six probes were designed for each gene and three biological replicates were analysed. Offset data of each gene were removed firstly based on criterion: 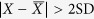 and average values were used to indicate transcriptional changes. Microarray data were deposited in NCBI (GEO number GSE64645).

### Construction of *gus* reporter plasmids

Promoters of *absA1*, *actII-orf4* and *cdaR* were amplified from genomic DNA with primers absA1pGR/absA1pGF, actII-orf4pGF/actII-orf4pGR, and cdaRpGF/cdaRpGR, respectively. Plasmid backbone was amplified from plasmid pLC-gus^6^ with primers pLCgusF/pLCgusR. Promoters were then assembled with plasmid backbone by Gibsion assembly to construct plasmids pLC-absA1p-gus, pLC-actII-orf4p-gus and pLC-cdaRp-gus. Plasmids were sequenced for validation and transformed into WT and ΔscbR2 for coloration detection^56^.

## Additional Information

**How to cite this article**: Li, X. *et al.* ScbR- and ScbR2-mediated signal transduction networks coordinate complex physiological responses in *Streptomyces coelicolor*. *Sci. Rep.* **5**, 14831; doi: 10.1038/srep14831 (2015).

## Supplementary Material

Supplementary Information

## Figures and Tables

**Table 1:** Targets of ScbR confirmed by EMSA.

Gene	Name	Fold change	Annotation	Possible binding site
sco1402-1403*	*cvnA4*	0.974	Putative large secreted protein	AGTAGTAGGCTCGCGCCGTTTGTTG
		0.544	Putative membrane protein	
sco1947*	*gap1*	0.650	Glyceraldehyde-3-phosphate dehydrogenase	ACGAACCGATCTCCTCGTTGGTAC
sco2373*	*tcmA*	0.906	Tetracenomycin C efflux protein	TTACTGACTCGTGAATTGGTTTGTCA
sco2907	*nagE2*	1.043	Putative PTS transmembrane component	TGGAAAAGACCGGATCCCCGCTTCTTT
sco3217	*cdaR*	0.420	Putative transcriptional regulator	GCCGCACCGCTGCGCAGGTTTGG
sco3867-3868	*soyB1*	0.611	Putative ferredoxin	CGCACTCCAGCGGCGGTCGGTTTCGG
		1.398	Putative uncharacterized protein	
sco4423	*afsK*	1.405	Serine/threonine-protein kinase AfsK	TCTTCCTGTCCGAGGCGCTGGTGGCGAACCT
sco4921*	*accA2*	1.650	Putative acyl-CoA carboxylase A subunit	CGTGCTGCGGGCCACGCGGTTTCTTT
sco4947*	*narG3*	1.487	Nitrate reductase alpha chain NarG3	GCCGACGCCGCTGACCGGCTTCTGA
sco5423*	*pyk2*	1.017	Pyruvate kinase	TTTTCGAACGGCGCATGGGTGCCATCCT ATCGGTTTGTTT
sco6268		1.874	Putative histidine kinase	TTAACAAACCTCCTCGCCGGTTTTCAAT
sco6312	*cprA*	0.969	Transcriptional regulator	AAAAACAGGCACACGGTCTGTTG
sco6323-6324		1.875	Putative tetR-family regulatory protein	ACTGAAAAGGGTTATTGCCTGTTTTGT
		0.974	Putative hydrolase	

^*^indicated targets obtained from ScbR2 targets. Divergent genes are listed in separated lines. Fold change value is the average of three biological duplicates.

**Table 2:** Targets of ScbR2 confirmed by EMSA.

Gene	Name	Fold change	Annotation	Possible binding site
sco1346–1347	*fabG3*	0.970	Putative 3-oxoacyl-ACP reductase	CTAGAAGCCCTGGCACCCGGTGTCA
		1.128	Putative secreted protein	
sco1402–1403	*cvnA4*	1.063	Putative large secreted protein	AGTAGTAGGCTCGCGCCGTTTGTTG
		0.747	Putative membrane protein	
sco1505	*rpsD*	1.418	30 S ribosomal protein S4	TGACAAGCCGGAAACCCAGAAAAGAGA
sco1570–1571	*argH*	1.161	Argininosuccinate lyase	TGTCTAACGATTATGCGGGTGCGG
		1.178	Putative uncharacterized protein	
sco1697–1698	*soxR*	1.088	Putative merR-family regulator	TCGCTCACCCGGTGCGCTCGTTTCTAAG
		1.637	Putative uncharacterized protein	
sco1947	*gap1*	0.768	Glyceraldehyde-3-phosphate dehydrogenase	ACGAACCGATCTCCTCGTTGGTAC
sco2373	*tcmA*	1.325	Tetracenomycin C efflux protein	TTACTGACTCGTGAATTGGTTTGTCA
sco2528–2529	*leuA*	0.982	2-isopropylmalate synthase	GGTCACGCGGGTCCGTATCAGT
		1.004	Putative metalloprotease	
sco2615–2616	*valS*	0.806	Folylpolyglutamate synthase	CCCGAAACG CGTTTCTTC
		1.455	Putative membrane protein	
sco2879	*cvnA12*	1.366	Putative uncharacterized protein	GGTCCTGGTAGTGGCTCAGTCGGTGT
sco2907	*nagE2*	1.099	Putative PTS transmembrane component	TGGAAAAGACCGGATCCCCGCTTCTTT
sco3067–3068		0.904	Putative anti anti sigma factor	CGCACACCGCAGTGCACGTATTTG
	*sig15*	0.953	RNA polymerase sigma factor	
sco3217	*cdaR*	0.756	Putative transcriptional regulator	GCCGCACCGCTGCGCAGGTTTGG
sco3224-3225		1.009	Putative ABC transporter ATP-binding protein	CGACGAATCGAATCGCTTGTAC
	*absA1*	0.912	Two component sensor kinase	
sco3229–3230		0.410	Putative 4-hydroxyphenylpyruvic acid dioxygenase	TTCGTTTTGCATTGTGAGGAGACAGGTGT
	*cdaPSI*	0.341	CDA peptide synthetase I	
sco3249		0.379	Putative acyl carrier protein	TTCGAACCTGCGACACCCGCTTTAGG
sco3615–3616	*ask*	1.010	Aspartokinase (EC 2.7.2.4)	GCTCCTCGCTCAATCCGTCTCTTT
		1.115	Putative uncharacterized protein	
sco3867–3868*	*soyB1*	0.613	Putative ferredoxin	CGCACTCCAGCGGCGGTCGGTTTCGG
		2.278	Putative uncharacterized protein	
sco3961	*serS*	0.947	Serine—tRNA ligase	AGGCCACCCTTCGTCCACCTGTTTCTTG
sco4035	*sigF*	1.077	RNA polymerase sigma-F factor	TTGCACACAGTGGACATGTCTTGTGA
sco4118	*atrA*	0.563	Putative tetR-family transcriptional regulator	ACGCACCCGGCGCTTGCGTTTGTCC
sco4423*	*afsK*	1.110	Serine/threonine-protein kinase AfsK	TCTTCCTGTCCGAGGCGCTGGTGGCGAACCT
sco4503		1.103	Putative long-chain-fatty acid CoA ligase	CAGCGACAGCAGAAGCAGTGTCTTT
sco4659	*rpsL*	1.871	30 S ribosomal protein S12	TAGGCACTACTTCTCCGGTTTCTGT
sco4677		1.550	Putative regulatory protein	GACGGACGCGGTGAGTTCGGTGGTGG
sco4921	*accA2*	2.464	Putative acyl-CoA carboxylase complex A subunit	CGTGCTGCGGGCCACGCGGTTTCTTT
sco4947	*narG3*	2.347	Nitrate reductase alpha chain NarG3	GCCGACGCCGCTGACCGGCTTCTGA
sco5085	*actII-orf4*	0.250	Actinorhodin operon activatory protein	ATAACAGGCCTACTCAACAGATTTCAAT
sco5216	*sigR*	1.620	RNA polymerase sigma factor	AGTGAGACCGGTCTCGGTTTCACG
sco5423	*pyk2*	0.783	Pyruvate kinase	TTTTCGAACGGCGCATGGGTGCCATCCTATCGGTTTGTTT
sco5544–5545	*cvnA1*	1.024	Putative membrane protein	GGAATGATGCCTTCAGGTGTGCAA
		0.952	Putative uncharacterized protein	
sco5881	*redZ*	0.449	Response regulator	GACGACCCGTGTCCTGGTGTGCTG
sco6060	*murC*	0.992	UDP-N-acetylmuramate—L-alanine ligase	ACAAGGTCGGCGTGCCGGTCCTGAA
sco6071	*cprB*	0.777	A-factor receptor homolog	ACTCAGAGCAGTTCGCTGGTCACTTG
sco6268		10.927	Putative histidine kinase	TTAACAAACCTCCTCGCCGGTTTTCAAT
sco6271–6272		N/A	Putative acyl-CoA carboxylase complex A subunit	ACATTTCCTTCTCTCTTGTTCTCA
		16.275	Putative secreted FAD-binding protein	
sco6275–6276		25.435	Putative type I polyketide synthase	GACTGATCACCTACCCGGTGTTTCT
		21.160	Putative secreted protein	
sco6282–6283		8.899	Putative 3-oxoacyl-ACP reductase	CTGCAATTACCCTCGGCGGTATGACG
		9.556	Putative uncharacterized protein	
sco6288		40.447	Putative regulatory protein	GAAGAGACCGAGCGGTCCGTTTCATT
sco6312	*cprA*	0.801	Transcriptional regulator	AAAAACAGGCACACGGTCTGTTG
sco6323–6324		0.923	Putative tetR-family regulatory protein	ACTGAAAAGGGTTATTGCCTGTTTTGT
		1.082	Putative hydrolase	
sco7623		0.904	NAD(P) transhydrogenase alpha subunit	CGAGAGACGGCCGTCGTTCTTG

^*^indicated targets obtained from ScbR targets. Divergent genes are listed in separated lines. Fold change value is the average of three biological duplicates.
