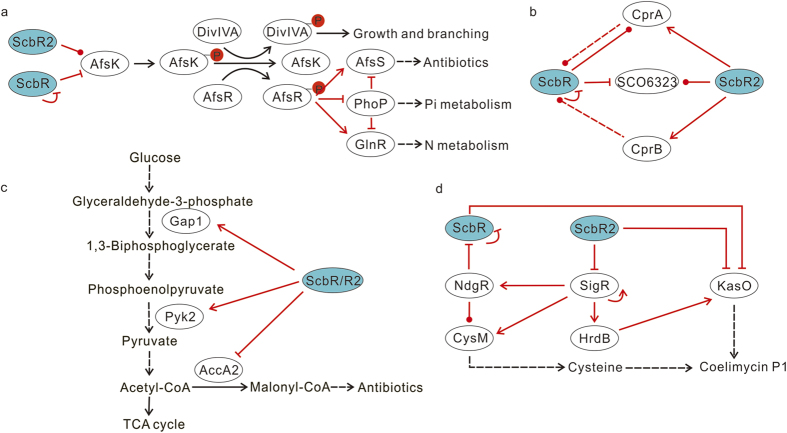# Corrigendum: ScbR- and ScbR2-mediated signal transduction networks coordinate complex physiological responses in *Streptomyces coelicolor*

**DOI:** 10.1038/srep21574

**Published:** 2016-03-04

**Authors:** Xiao Li, Juan Wang, Shanshan Li, Junjie Ji, Weishan Wang, Keqian Yang

Scientific Reports 5: Article number: 14831; 10.1038/srep14831 published online: 10072015; updated: 03042016.

This Article contains errors in Figure 4.

In Figure 4c, the enzyme Pyk2 was misplaced and Pyruvate was omitted from the reaction.

In Figure 4d, the arrow between ScbR2 and SigR should represent repression of activity.

The correct Figure 4 appears below as Fig. 1.

## Figures and Tables

**Figure 1:**