# Supplementary material for: ScbR- and ScbR2-mediated signal transduction networks coordinate complex physiological responses in Streptomyces coelicolor
Source: Sci Rep. 2015 Oct 7;5:14831. doi: 10.1038/srep14831 (PMC4595836; doi:10.1038/srep14831)
Supplement: Supplementary Information [file srep14831-s1.doc]

**ScbR- and ScbR2-mediated signal transduction networks coordinate complex physiological responses in *Streptomyces coelicolor***

Xiao Li, Juan Wang, Shanshan Li, Junjie Ji, Weishan Wang*, Keqian Yang*

**Supplementary Figure S1.** **Phenotypes and antibiotic production profiles of ΔscbR and ΔscbR2 compared to M145.**

**
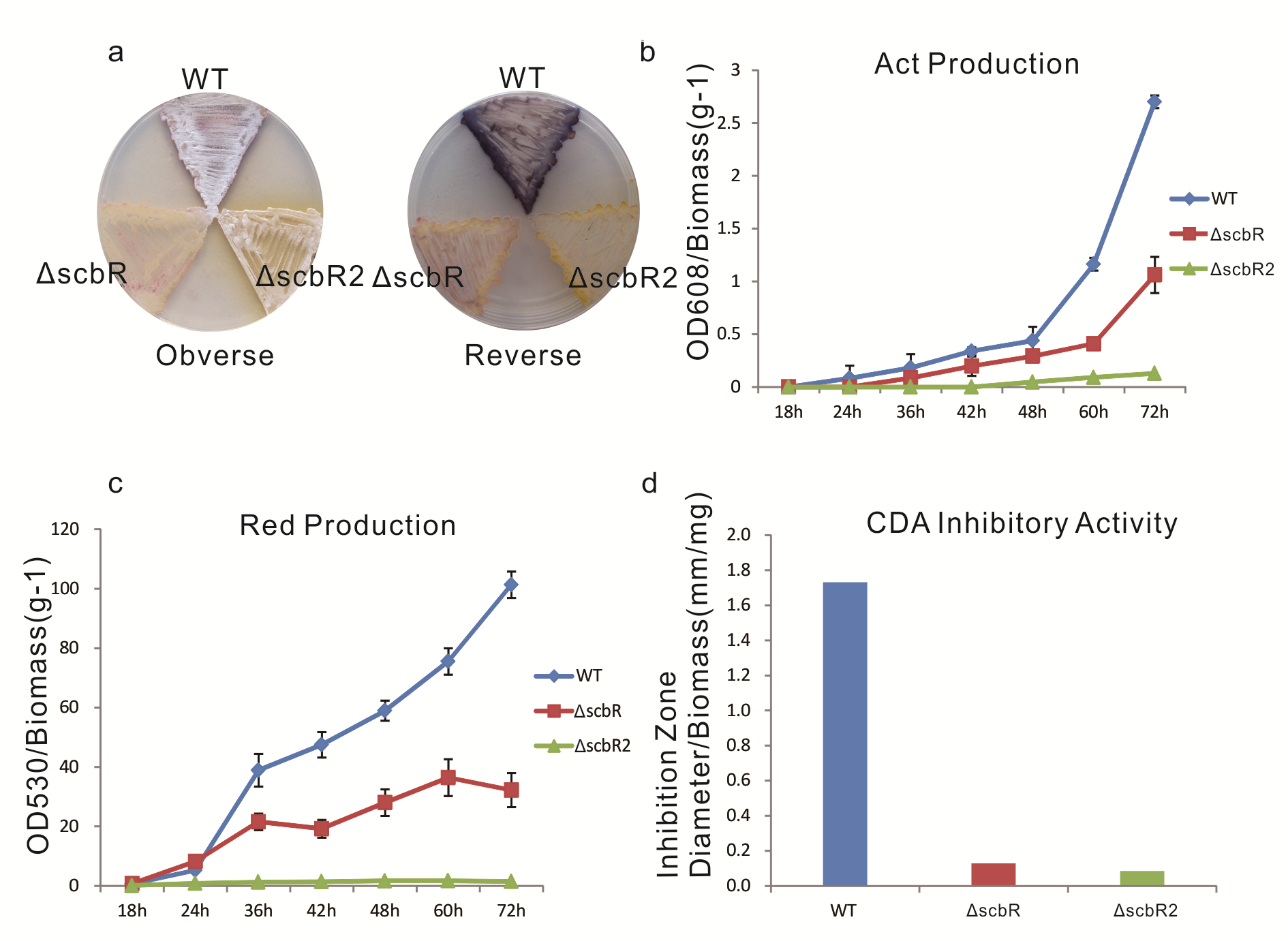
**

(a) Phenotypes and antibiotics production of ΔscbR, ΔscbR2 and M145 on SMMS plates. Spores of ΔscbR, ΔscbR2 and M145 were incubated on SMMS plates at 30 °C; growth status was photographed after 36 hour while antibiotic production was after 3 days. (b, c, d) Antibiotics production profiles in liquid SMM culture, CDA production was measured at 72h.

**Supplementary Figure S2. Analysis of distribution of binding sites of ScbR and ScbR2 on the genome.**


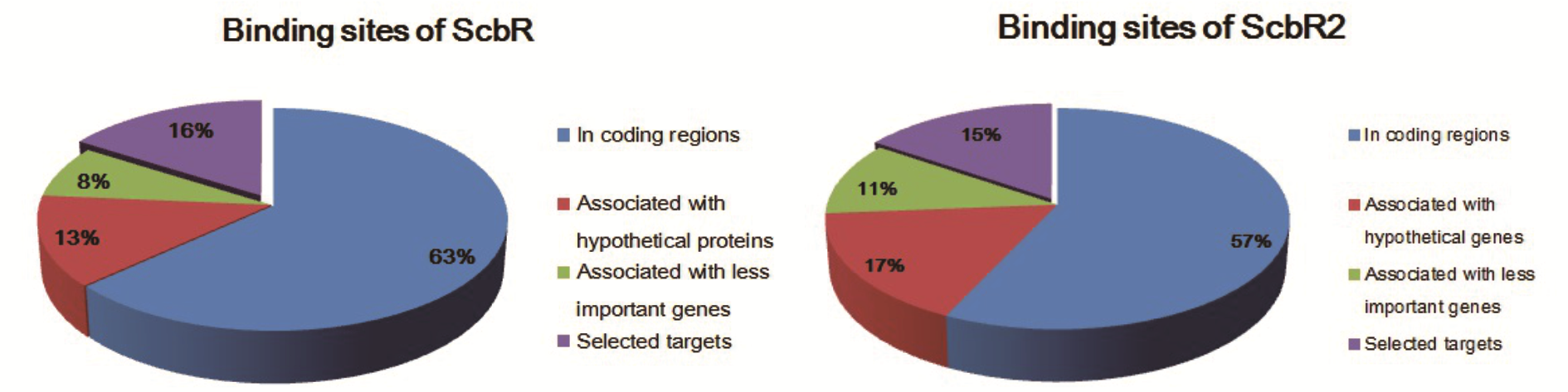


Binding sites locates in the coding regions, associated with hypothetical genes, and with less important genes were not considered, but targets associated with genes that were studied or with important annotations were selected in this work.

**Supplementary Figure S3. EMSA assays of purified ScbR and ScbR2 with selected promoters.**


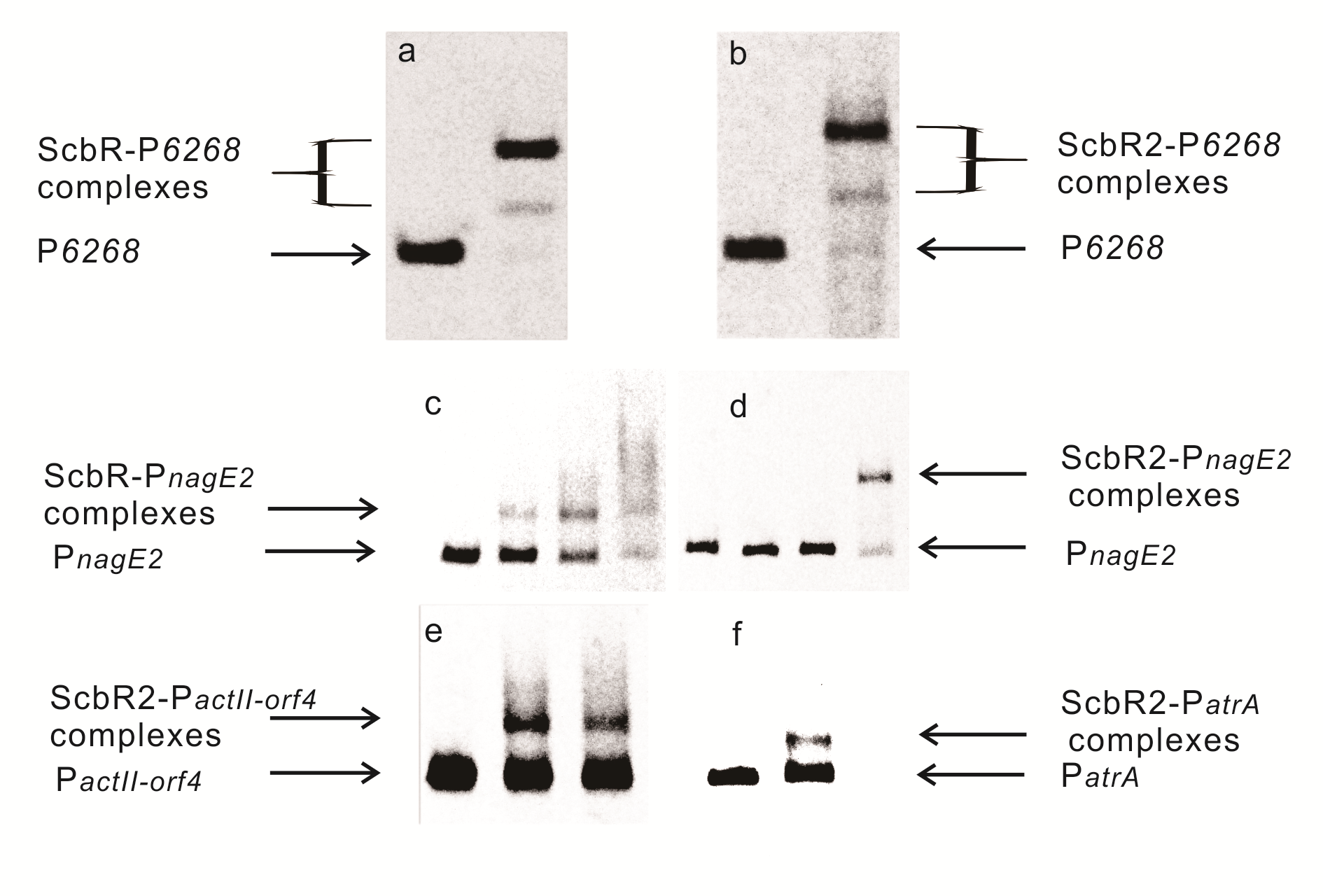


Each lane contained 6 ng probe P*6268,* and 0.21 μM ScbR (a) or 0.31 μM ScbR2 proteins (b). Each lane of (c) and (d) contained 6 ng probes, purified ScbR protein of 0, 0.377, 1.26, 3.77 μM (c), or ScbR2 protein of 0, 0.207, 0.621, 2.07 μM (d). Each lane of (e) contained 12 ng probes and ScbR2 protein of 0, 1.245, 4.15 μM. Each lane of (f) contained 10 ng probes and ScbR2 protein of 0 and 0.64 μM.

**Supplementary Figure S4.** **Evaluation the effects of ScbR2 on *absA1*, *actII-orf4* and *cdaR* promoters by Gus reporter gene.**


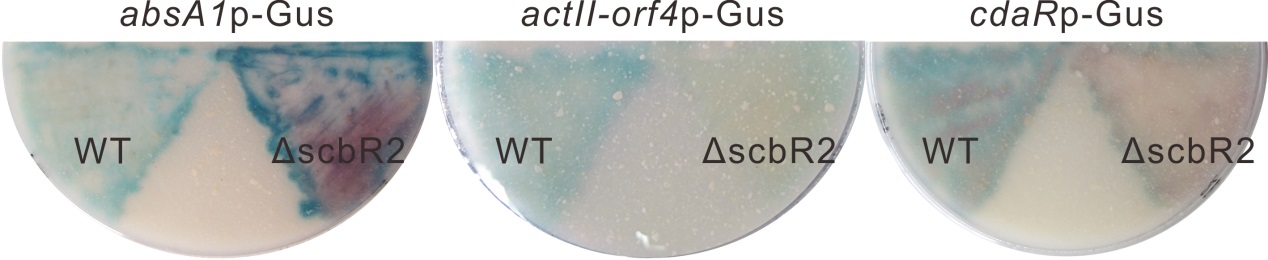


Activities of *absA1*, *actII-orf4* and *cdaR* promoters were measured in WT and ΔScbR2, reported by *gus* reporter gene. *absA1* was repressed while *actII-orf4* and *cdaR* were activated by ScbR2.

**Supplementary Figure S5. Peaks of ScbR2 upstream and inside of rRNA genes.**


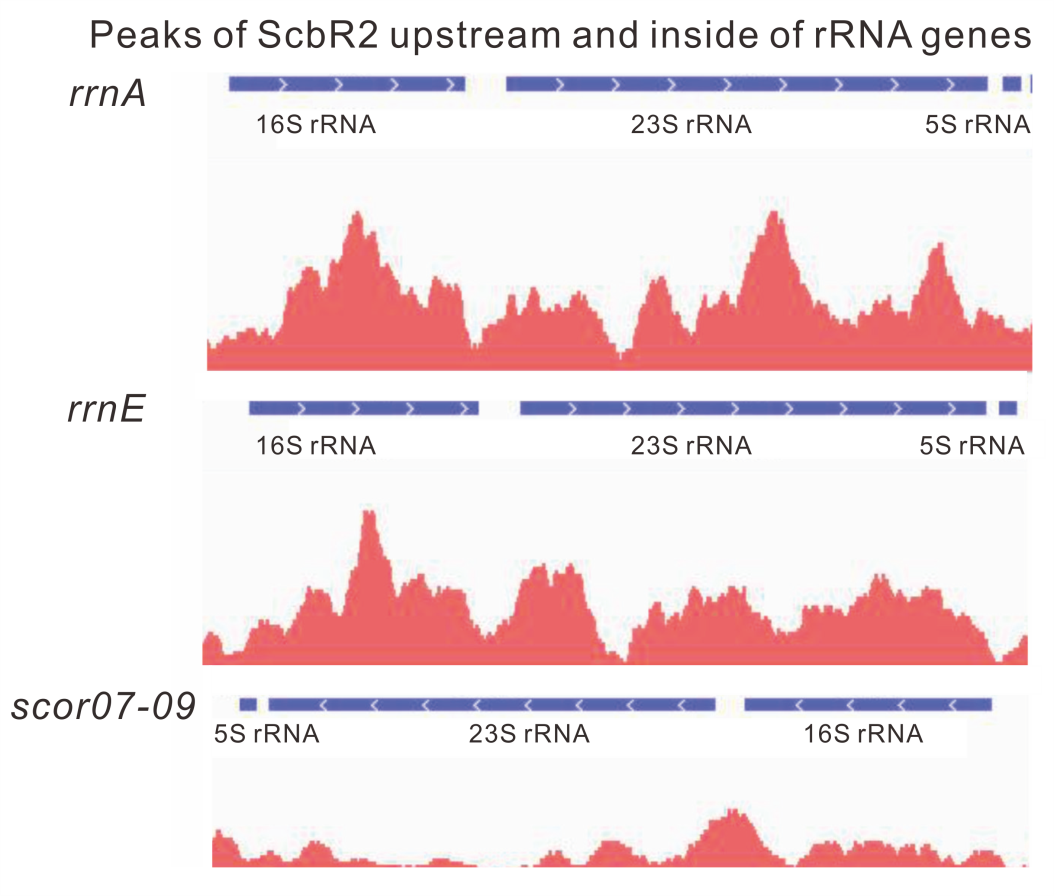


Peaks of ScbR2 were observed located inside of rRNA gene *rrnA* and *rrnE*, which comprise 16s, 23s and 5s ribosomal RNA genes, and in front of the promoter region of *scor08* on ScbR2 ChIP-seq map, implying involvement of ScbR2 on rRNA synthesis.

**Supplementary Table S1. Targets of ScbR selected for EMSA assays.** 23 targets of ScbR were selected to carry out EMSA assays. Their ID, names and annotation were listed, and genes were arranged by their IDs.

| **Gene** | **Name** | **Annotation** |
| --- | --- | --- |
| sco2156 | *cox* | Probable cytochrome c oxidase subunit 2 |
| sco2158 |  | Putative kinase |
| sco2736 | *citA* | Citrate synthase |
| sco2777 | *accC* | Acetyl/propionyl CoA carboxylase alpha subunit |
| sco2792 | *adpA* | AraC-family transcriptional regulator |
| sco2905 |  | Putative uncharacterized protein SCO2905 |
| sco2907 | *nagE2* | Putative PTS transmembrane component |
| sco3034 | *whiB* | Transcriptional regulator WhiB |
| sco3337 | *proC* | Pyrroline-5-carboxylate reductase |
| sco3571 | *crp* | Putative transcriptional regulator |
| sco3867 | *soyB1* | Putative ferredoxin |
| sco3926 | *ssgA* | Putative regulator |
| sco4423 | *afsK* | Serine/threonine-protein kinase AfsK |
| sco4426 | *afsR* | Regulatory protein AfsR |
| sco5231 | *dasR* | HTH-type transcriptional repressor DasR |
| sco5357 | *rho* | Transcription termination factor Rho |
| sco5848 | *agaZ* | Tagatose 6-phosphate kinase |
| sco5852 | *agaY* | Tagatose-bisphosphate aldolase |
| sco5893 | *redK* | Oxidoreductase |
| sco6071 | *cprB* | A-factor receptor homolog |
| sco6108 | *fusH* | Esterase |
| sco6312 | *cprA* | Transcriptional regulator |
| sco6323 |  | Putative tetR-family regulatory protein |

**Supplementary Table S2. Targets of ScbR2 selected for EMSA assays.** 76 targets of ScbR2 were selected to carry out EMSA assays. Their ID, names and annotation were listed, and genes were arranged by their IDs.

| **Gene** | **Name** | **Annotation** |
| --- | --- | --- |
| sco0216 | *narG2* | Nitrate reductase alpha chain NarG2 |
| sco0384 |  | Putative membrane protein |
| sco0498 |  | Putative peptide monooxygenase |
| sco0596 |  | DpsA (Putative DNA-binding protein) |
| sco1345 | *fabG2* | Putative 3-oxoacyl-ACP reductase |
| sco1346 | *fabG3* | Putative 3-oxoacyl-ACP reductase |
| sco1402 | *cvnA4* | Putative large secreted protein |
| sco1505 | *rpsD* | 30S ribosomal protein S4 |
| sco1570 | *argH* | Argininosuccinate lyase |
| sco1630 | *cvnA9* | Putative integral membrane protein |
| sco1697 | *soxR* | Putative merR-family transcriptional regulator |
| sco1712 |  | Putative TetR-family transcriptional regulator |
| sco1947 | *gap1* | Glyceraldehyde-3-phosphate dehydrogenase |
| sco2373 | *tcmA* | Tetracenomycin C efflux protein |
| sco2486 | *nirB* | Putative nitrite reductase NirB |
| sco2528 | *leuA* | 2-isopropylmalate synthase |
| sco2615 | *valS* | Valine--tRNA ligase |
| sco2782 |  | Putative pyridoxal-dependent decarboxylase |
| sco2879 | *cvnA12* | Putative membrane protein |
| sco2907 | *nagE2* | Putative PTS transmembrane component |
| sco3068 | *sig15* | RNA polymerase sigma factor |
| sco3201 |  | Putative tetR-family transcriptional regulator |
| sco3217 | *cdaR* | Putative transcriptional regulator |
| sco3218 |  | Putative uncharacterized protein SCO3218 |
| sco3225 | *absA1* | Two component sensor kinase |
| sco3226 | *absA2* | Two component system response regulator |
| sco3229 |  | Putative 4-hydroxyphenylpyruvic acid dioxygenase |
| sco3230 | *cdaPSI* | CDA peptide synthetase I |
| sco3245 |  | Putative salicylate hydroxylase |
| sco3248 | *fabF3* | Putative 3-oxoacyl-ACP synthase II |
| sco3249 |  | Putative acyl carrier protein |
| sco3615 | *ask* | Aspartokinase |
| sco3961 | *serS* | Serine--tRNA ligase |
| sco4008 |  | Putative tetR family regulatory protein |
| sco4035 | *sigF* | RNA polymerase sigma-F factor |
| sco4118 | *atrA* | Putative tetR-family transcriptional regulator |
| sco4503 |  | Putative long-chain-fatty acid CoA ligase |
| sco4635 | *rpmG3* | 50S ribosomal protein L33 2 |
| sco4659 | *rpsL* | 30S ribosomal protein S12 |
| sco4677 |  | Putative regulatory protein |
| sco4921 | *accA2* | Putative acyl-CoA carboxylase complex A subunit |
| sco4947 | *narG3* | Nitrate reductase alpha chain NarG3 |
| sco5059 | *ppgK* | Polyphosphate glucokinase |
| sco5085 | *actII* | Probable actinorhodin operon activatory protein |
| sco5086 | *actIII* | Putative ketoacyl reductase |
| sco5087 | *actIORF1* | Actinorhodin polyketide putative beta-ketoacyl synthase 1 |
| sco5216 | *sigR* | RNA polymerase sigma factor |
| sco5222 |  | Epi-isozizaene synthase |
| sco5316 |  | Probable acyl carrier protein (ACP) (WhiE ORF V) |
| sco5319 |  | 16.7 kDa protein in whiE locus (WhiE ORF II) |
| sco5423 | *pyk2* | Pyruvate kinase |
| sco5544 | *cvnA1* | Putative membrane protein SC1C2.25c |
| sco5881 | *redZ* | Response regulator |
| sco5882 | *redV* | RedV protein |
| sco5897 | *redG* | Putative oxidase |
| sco5898 | *redF* | Probable membrane protein |
| sco5998 | *murA2* | Putative UDP-N-acetylglucosamine transferase |
| sco6060 | *murC* | UDP-N-acetylmuramate--L-alanine ligase |
| sco6071 | *cprB* | A-factor receptor homolog |
| sco6106 |  | Putative acyl-CoA dehydrogenase |
| sco6267 |  | Putative uncharacterized protein SCO6267 |
| sco6268 |  | Putative histidine kinase |
| sco6271 | *accA1* | Putative acyl-CoA carboxylase complex A subunit |
| sco6272 |  | Putative secreted FAD-binding protein |
| sco6275 |  | Putative type I polyketide synthase |
| sco6278 |  | Putative integral membrane transport protein |
| sco6282 |  | Putative 3-oxoacyl-ACP reductase |
| sco6283 |  | Putative uncharacterized protein SCO6283 |
| sco6286 | *scbR2* | Putative regulatory protein |
| sco6287 | *scoT* | Thioesterase II |
| sco6288 |  | Putative regulatory protein |
| sco6312 | *cprA* | Transcriptional regulator |
| sco6323 |  | Putative tetR-family regulatory protein |
| sco7056 |  | Putative gntR-family transcriptional regulator |
| sco7463 | *cvnA13* | Putative sensor histidine kinase |
| sco7623 |  | NAD(P) transhydrogenase alpha subunit |

**Supplementary Table S3**. Strains and plasmids used in this study.

| **Bacterial strains** | **Relevant genotypes** | **Source/Reference** |
| --- | --- | --- |
| *S. coelicolor* |  |  |
| M145 | Plasmid-free derivative of *S. coelicolor* A(3)2 | [^1^](#_ENREF_1) |
| ΔscbR2 | The scbR2 disruption mutant | [^2^](#_ENREF_2) |
| ΔscbR | The scbR disruption mutant | This study |
| *E. coli* |  |  |
| JM109 | General cloning host for plasmid manipulation | Novagen |
| BL21(DE3) | Host for expression plasmids with T7 derived promoter | Novagen |
| ET12567(pUZ8002) | Donor strain for conjugation between *E. coli* and *Streptomycetes* | [^1^](#_ENREF_1) |
| Plasmids |  |  |
| pET23b | Protein expression plasmid, amp^R^ | Novagen |
| pET23b-ScbR | Expression of his_6_-ScbR, amp^R^ | [^2^](#_ENREF_2) |
| pET23b-ScbR2 | Expression of his_6_-ScbR2, amp^R^ | [^2^](#_ENREF_2) |
| pKC1139 | Replicative vector, aac(3)IV, pSG5 origins of replication | [^3^](#_ENREF_3) |
| pKC1139-∆scbR | Disruption plasmid for scbR gene disruption | This study |
| pLC-gus | Integrated vector, hyg^R^, carrying a promoterless gusA reporter gene | [^4^](#_ENREF_4) |
| pLC-absA1p-gus | pLCgus with *absA1* promoter inserted in front of gusA | This study |
| pLC-actII-orf4p-gus | pLCgus with *actII-orf4* promoter inserted in front of gusA | This study |
| pLC-cdaRp-gus | pLCgus with *cdaR* promoter inserted in front of gusA | This study |

**Supplementary Dataset S1. Oligonucleotide primers used in this study. Restriction sites in the primers were shown.**

| **Name** | **Sequence** | **Description** |
| --- | --- | --- |
| scbRLarmF | AAAA AAGCTT GGGCACCCCTGCAAGCAC | Used to amplify the left homologous arm of s*cbR* gene |
| scbRLarmR | AAAA GGATCC ACCGGGAAGGACTGAC |
| ScbRRarmF | AAAA GGATCC AGTGGAAGTAGAGGGCTC | Used to amplify the right homologous arm of s*cbR* gene |
| ScbRRarmR | AAAAA TCTAGA GCTCGGTGGCACTGTTCG |
| sco6268pR-foot | HEX-CCCTCATGTCCGAACAGCTCGACA | Used to amplify HEX-lablled *sco6268* promoter |
| sco6268pF-foot | CTGAGCCCGTCCTGCAACGTCTCCGTG |
| absA1pGR | AGACCGGTCATCTTGTTGCCTCCTTAGCA  GTCCAGCACGCTACAAGAGCGGCCC | Used to amplify absA1p for Gibsion assemblling |
| absA1pGF | TCGTCATCGTCAAGTGTTAATAGGGTCTT  CTGCGCATCGTCGTCGACTTTCCCG |
| actII-orf4pGR | AGACCGGTCATCTTGTTGCCTCCTTAGCA  GCTGCGCCCCCGTCGAGATTCTCCG | Used to amplify actII-4p for Gibsion assemblling |
| actII-orf4pGF | TCGTCATCGTCAAGTGTTAATAGGGTCTT  CTGCTGATCGCGAGCGTGGTGCTGT |
| cdaRpGR | AGACCGGTCATCTTGTTGCCTCCTTAGCAG  GACCTCTTGATACCCCCGCGTCAA | Used to amplify cdaRp for Gibsion assemblling |
| cdaRpGF | TCGTCATCGTCAAGTGTTAATAGGGTCTT  CGGGCTGGTGCTGGAGTTGGTGTGG |
| pLC-gusF | CAGTGGCAACTAAGTACAATTAAAGGCT | Used to amplify pLC-gus plasmid for Gibsion assembling |
| pLC-gusR | CTGCTAAGGAGGCAACAAGATGACCGGT |
| SCO2156pF | AAGTCCTCGTATGTGCAACCGGTG | Primers used to amplify promoter regions of ScbR targets |
| SCO2156pR | CCCTCGTCCAGAGACGCCAACA |
| SCO2158pF | TGTGCAGCTGGTCCGCGACGAG |
| SCO2158pR | GCCAGGTCGCCGTCCAGGTCGG |
| SCO2736pF | GCTTCCCGATGTCGAAGCCCTTGT |
| SCO2736pR | TACGACTTCGACGAACTCGATGAAC |
| SCO2777pF | TGCAGAACATCTCCGGGTTCATGG |
| SCO2777pR | GTCGGAGAAGACCGCCACCGAG |
| SCO2792pF | CCGGCAGCGGGCGCGCGGACG |
| SCO2792pR | TTGCGGCGTCGCCCGGACAGC |
| SCO2905pF | TTCGTTGACCAGCGCGGGGTCG |
| SCO2905pR | AGAATGCCGGCGGCGGGCAGGACC |
| SCO2907pF | CGATCACGAAGTTCGATCTGAAGA |
| SCO2907pR | CGATCGGGAGCTGGAGGCTGCGTC |
| SCO3034pF | TCGGGGAAGAAGGACTCGGGGTCG |
| SCO3034pR | CCAAGTTCGCGAGCATGCCCCCAAG |
| SCO3337pF | TGAGCAGGGCTTCGCCGATCTTG |
| SCO3337pR | TCGGAATTCCAGGCGGTCCAGTTC |
| SCO3571pF | CGCTCTTCTTCGCGTCGGACTT |
| SCO3571pR | AGGGTGTCGCCGCGGGCGAGGGTC |
| SCO3867pF | TATGACGGCGCCGAGGCCGCCCA |
| SCO3867pR | TCCACGTACTCCCCGTAGGCGT |
| SCO3926pF | CACGATGGCCATCTCACTGC |
| SCO3926pR | TCTCCGGGCAGATGAAAGGTCAG |
| SCO4423pF | CGGAGGCCGCCAACGCCTTCTA |
| SCO4423pR | GACGCCGAGCGCGCGAGATAGACCA |
| SCO4426pF | AGCGTCTCCCCGTCCCGCCAGGC |
| SCO4426pR | TTTCGGGCCTCCCCCGCCGCACA |
| SCO5231pF | GTCTGGGTCCGGGTCATGTCGAG |
| SCO5231pR | GATCGAGACCATCATGCCCGCGATA |
| SCO5357pF | TGGACCAGCAGGGCGCGACCGTCCT |
| SCO5357pR | AGCACCATGCCCTCGAGGCCGGTA |
| SCO5848pF | ACTTCAGCACCGCCTCGCTGTAC |
| SCO5848pR | TGCAGCACGGCCGCCTCGATGAC |
| SCO5852pF | TCCGGGGCTCCCTGGCAGGCGC |
| SCO5852pR | CCTGCGCACCGGAGGCGATCGC |
| SCO5893pF | GGGCGAGCACTACGGCACCGAC |
| SCO5893pR | GCTCGGCCAGGCGGTGCACCAGGTG |
| SCO6071pF | GAGGGCGGAGCAGCCGGCCAC |
| SCO6071pR | CGTAGCCGCGGCGGTCGAACA |
| SCO6108pF | ACGACCGCGAACGGCGAGGGCGAC |
| SCO6108pR | GCTCGCCGGAAATCGCCTGCGCGG |
| SCO6312pF | TAGCCGTGGCGGTCGAACAGG |
| SCO6312pR | CGCCGCAACCGCGCACTGGCG |
| SCO6323pF | CGTTCTGTCCGTCCTCGTCTGTTCTGGTGC |
| SCO6323pR | TGGGCCTCCCATGCTTTCGCATGTATGCGG |
| SCO0216pF | TGTACGTGGACATGGCCACTCCTT | Primers used to amplify promoter regions of ScbR2 targets |
| SCO0216pR | AGAGGCCAGTCCCGGGCGA |
| SCO0384pF | AGGAGCGGGTCACCGGACGGCT |
| SCO0384pR | TCGACCACCGCCGAGCTGGTCGCTA |
| SCO0498pF | TGTGCGTTGTGTTCGCGGATCG |
| SCO0498pR | ACGGAGTCGCTCCAGATCTTCT |
| SCO0596pF | ACGTCATCAGCGAGATCGGC |
| SCO0596pR | TCAGGTGCAGGTCGTTGAGG |
| SCO1345pF | TAGCCGATGCCGCGGCTCGC |
| SCO1345pR | AACGCCATCGCCTTCTTCA |
| SCO1346pF | ATGCCGCGCGCGGCTCCGGTGACTA |
| SCO1346pR | ACGATCGCGGCGATCGCGACTGCTAT |
| SCO1402pF | AGATCGCGGTCAGGGACACCA |
| SCO1402pR | ACCGCCGAGGGCTCCTCGG |
| SCO1505pF | ATGCCGAGGGCACGCGACTT |
| SCO1505pR | TGCCCTGGACGACGTGCGCAA |
| SCO1570pF | CGAAACGGCCGCCCCAGA |
| SCO1570pR | TTGGGGGACAGGTTGACCGT |
| SCO1630pF | AACAGCGTTCGCGCGTAGGGAAGTT |
| SCO1630pR | ACTTCGTGCGCTGGTGCAAGCAGGT |
| SCO1697pF | GCGCGTCACGGGCGAAGCGCCGC |
| SCO1697pR | CGGGTCCACGTGGATCCACTGGTGG |
| SCO1712pF | GGAGGTCGGCGCAGGCGTCGAGGAT |
| SCO1712pR | GCCTGCCGGCCGGCCTGGTCCCGC |
| SCO1947pF | GGAAGTAGTTACGACCGATG |
| SCO1947pR | ACGGACGACACCGTGCTGCT |
| SCO2373pF | GCGCCATCCCTTGGCGGGCGCCGA |
| SCO2373pR | AGGCCGCGGGTGCGGTCT |
| SCO2486pF | ACCACGGCGTCGATCCAGC |
| SCO2486pR | TCACCGAGCCGCCGGGCGAGCGT |
| SCO2528pF | TCGGCGATGTCGACCTGGTCGTA |
| SCO2528pR | TGTGGCGTCATGTCCGGCACT |
| SCO2615pF | TCTACCTCGGCCGGCGTGTACT |
| SCO2615pR | TAGCCGTAGCCGGGCTGGGGA |
| SCO2782pF | GTGAAGCGGCTGCGGCGGCACTT |
| SCO2782pR | TCGATGCGGGGGGAGAGGGCGTC |
| SCO2879pF | TCCGACACCGACTGAGCCACTA |
| SCO2879pR | AAGTACCACGCGGCCCCGGCT |
| SCO3068pF | ACTGGGCACTGCCGACCGT |
| SCO3068pR | GATCCAGATGTTCCGGAGGGAGT |
| SCO3201pF | GGGTCTCGGCCTTGCGCCGCTCGG |
| SCO3201pR | GAGCACGGCCTCACCCGCGAGCGC |
| SCO3217pF | GGGCTGGTGCTGGAGTTGGTGTGG |
| SCO3217pR | AGTGGCCCGAGCACTCCGAATT |
| SCO3218pF | ATCGTTGACCAGCACGAGATA |
| SCO3218pR | CACCTGTCCTTCACCGACTA |
| SCO3224-S25pF | TGCGCATCGTCGTCGACTTT |
| SCO3224-25pR | CCGAACCACGGATAGAACAGC |
| SCO3226pF | GCACGACCTGCGAGTACTACCG |
| SCO3226pR | GCTTGCGGGCCAGTTCCACC |
| SCO3229-30pF | AAGCCGGAGGCCTCCCGGTCGTCC |
| SCO3229-30pR | GGATCCCGTCCGGTAGTGCGCGCC |
| SCO3245pF | GCGTACCTCGTGGCCCTTCCGGGT |
| SCO3245pR | TGGTGTCGAACATCGCGGACGT |
| SCO3248pF | GTCACCGCCCTCCAGGGACC |
| SCO3248pR | CAAGGAGGAGACCGGCGTGG |
| SCO3249pF | TCGGCCAGAATCCCGTGCACAAC |
| SCO3249pR | GCGCCCCGTGTCGTGATGGAGATC |
| SCO3615pF | TTGATGCCCTCGGCATCGGCTA |
| SCO3615pR | AGGCATCCAGGACGCCGATCA |
| SCO3961pF | TCTCCACGGGCGCGCTGCGA |
| SCO3961pR | AGTCGACATATCCGCCTACAGTT |
| SCO4008pF | AAGAGCCGGGCGGCGGCGCCGGAC |
| SCO4008pR | TGCGGTCGATGCGCGCGCCGGCGAT |
| SCO4035pF | GGGTGTCGGCGCCCCGGCTGCGCT |
| SCO4035pR | GACGACGCGGACGCGCTCAAGCGCT |
| SCO4118pF | TACTCATCGCACCGCCGGGCGCGA |
| SCO4118pR | AGTTCGCCCTTGAGATTCCGTT |
| SCO4503pF | CCTGATCTCGATCTGCGC |
| SCO4503pR | GAGTCGGCGTAGGCGGCGGAT |
| SCO4635pF | TGTGTCACCGGTTCAAGT |
| SCO4635pR | AGGCCAGCGTGATCTTCG |
| SCO4659pF | TCCGCCGACACGCTCGCCGAACA |
| SCO4659pR | ACCTTGTCCTGCCGGCCCTTC |
| SCO4677pF | GAACGCAGCTGCTCGGTCGC |
| SCO4677pR | CCCGGCGGAGCTTCAACTGC |
| SCO4921pF | ACGCGGACAGCGATTTCGC |
| SCO4921pR | ACATGTCCTGGCGGTCGACGA |
| SCO4947pF | AAGCTGCCGGTGCGCATTCC |
| SCO4947pR | AGCAGCTGACCTGCCTTCAGAA |
| SCO5059pF | AGGAGTCGATCATCTTCT |
| SCO5059pR | AGGTCGCCCCTGTCGAGGTCCACA |
| SCO5085pF | ATCGTGCGATCGGTCCTGGT |
| SCO5085pR | GGAGCAGCAGCACCAGGAGC |
| SCO5086-87pF | ACGAACACGCGCAACCCCTC |
| SCO5086-87pR | TTCGTCCGGTGGTCGTCGTCAT |
| SCO5216pF | AGGTGCCGTACGGCGACCACGGCTT |
| SCO5216pR | TGCGTCGGTCCCAGTGAC |
| SCO5222pF | TGTTTGCAGCACGAGACGGAC |
| SCO5222pR | TTGTCTCCTGGAGCTTCGGCCAAT |
| SCO5316pF | AAGGTGTCGAAACCGTCGTCG |
| SCO5316pR | ACCCCGCACGTGCTGGACGTGT |
| SCO5319pF | ATGAAGCCGCTGGTCGACCCGG |
| SCO5319pR | AGGTCCTCGGCCTGCACGGCCG |
| SCO5423pF | ATCAGCGTGACGAGCTGCTCG |
| SCO5423pR | TGGTACCGACGGACGAGGAAAT |
| SCO5544pF | TCCGCCGAAGCGCTGCCATC |
| SCO5544pR | GGTTTTGCCTTCTCTGTAAAG |
| SCO5881pF | TGGTCTCCACCTGCACTTTCATGT |
| SCO5881pR | CCACCGGCGCGCGGACGCCGCCGT |
| SCO5882pF | TTGTGGGAGGAGGGACTCAG |
| SCO5882pR | TACTACGGCTTCGGACTGCG |
| SCO5897pF | CGCATCAACACCGGCGACCTGATC |
| SCO5897pR | CGCCAGAGCACGAGGTCCTGGCCCAT |
| SCO5898pF | CCTCGGCCGCGCCCTCGTCACCGG |
| SCO5898pR | CGCCGCCGAACAGGAACCACACGGC |
| SCO5998pF | AGCTGTGACTGCGTCCAGCC |
| SCO5998pR | CTAAGTTAGGTAACCCTTAC |
| SCO6060pF | ATCGTCCTGACCGACCCCCACTA |
| SCO6060pR | ATGCCGATGAAGTGCGGTCGGT |
| SCO6071pF | GAGGGCGGAGCAGCCGGCCAC |
| SCO6071pR | CGTAGCCGCGGCGGTCGAACA |
| SCO6106pF | TGAAGACGTCGTAGCCGGTCA |
| SCO6106pR | GATGTTGTCGCGCAGGCTCTC |
| SCO6267pF | CCGGCGCCCTTCGTGCCGTCGATC |
| SCO6267pR | GTTCCGGCCGCCGGGCGAGGACTC |
| SCO6268pF | CGGGGAGCCCTGGATGTGCCGTGC |
| SCO6268pR | GCCTCGCCGACCCCGACACCCTGC |
| SCO6271-72pF | GATCCGCGTAGACGGCCACGCTCG |
| SCO6271-72pR | GCGGCGGGCGCGGCCGTCGCCGGC |
| SCO6275pF | GGGTCCGCGGCCCCGGGGAGCCGG |
| SCO6275pR | GGTCCACGACCGTGACGTTCGCG |
| SCO6278pF | GCCCTGAACTGGTACCGCGCCAAC |
| SCO6278pR | CAGGTCGGCGGTCATCAGCGTCAC |
| SCO6282-83pF | GCCGCTGGGCGATGGCACGCCCG |
| SCO6282-83pR | GGGAGGCCGGCCGCGCGGGCACGC |
| SCO6286-87pF | CATCGTGAGCGACGCGGGCGCGAAC |
| SCO6286-87pR | CGCGAGGAACGCTCCCGCGGCGCC |
| SCO6288pF | GTCACTTCTATCTCGACCAGAGCG |
| SCO6288pR | TCGTTCGGCCGGGTCAGCAGCAGG |
| SCO6312pF | TAGCCGTGGCGGTCGAACAGG |
| SCO6312pR | CGCCGCAACCGCGCACTGGCG |
| SCO6323pF | CGTTCTGTCCGTCCTCGTCTGTTCTGGTGC |
| SCO6323pR | TGGGCCTCCCATGCTTTCGCATGTATGCGG |
| SCO7056pF | AACTCGCCGAGCAGTTGGTC |
| SCO7056pR | GACTTCAACGATCACGAGGG |
| SCO7463pF | AGCCGACCACGCTGTTGATG |
| SCO7463pR | GACGAGGGCGACCCAGAGCAA |
| SCO7623pF | ACACGCGTCTCCCCGGGCAG |
| SCO7623pR | AAGGCCGTGCACGACGGCACG |

**References:**

1 Kieser, T. & Foundation, J. I. *Practical Streptomyces Genetics*. (John Innes Foundation, 2000).

2 Xu, G. *et al.* "Pseudo" gamma-butyrolactone receptors respond to antibiotic signals to coordinate antibiotic biosynthesis. *J Biol Chem* **285**, 27440-27448, doi:10.1074/jbc.M110.143081 (2010).

3 Bierman, M. *et al.* Plasmid cloning vectors for the conjugal transfer of DNA from Escherichia coli to Streptomyces spp. *Gene* **116**, 43-49 (1992).

4 Wang, W. *et al.* Angucyclines as signals modulate the behaviors of Streptomyces coelicolor. *Proceedings of the National Academy of Sciences of the United States of America* **111**, 5688-5693, doi:10.1073/pnas.1324253111 (2014).
